# REPLY BY THE AUTHORS: RE: Complete corporeal preservation clitoroplasty: new insights into feminizing genitoplasty

**DOI:** 10.1590/S1677-5538.IBJU.2021.0314.1

**Published:** 2021-05-30

**Authors:** Nicolas Fernandez, Julián Chavarriaga, Jaime Pérez

**Affiliations:** 1 Pontificia Universidad Javeriana Hospital Universitario San Ignacio Division of Urology Bogota Colombia Division of Urology, Hospital Universitario San Ignacio, Pontificia Universidad Javeriana, Bogota, Colombia.; 2 Fundacion Santa Fe de Bogota Department of Urology Bogota Colombia Department of Urology, Fundacion Santa Fe de Bogota, Bogota, Colombia.; 3 University of Washington Seattle Children's Hospital Division of Urology SeattleWA United States Division of Urology, Seattle Children's Hospital, University of Washington, Seattle, WA, United States.

*To the editor,*

We appreciate very much your comment about our paper published recently in Int Braz J Urol (1). In response to your concern about the similarity (2) to the Kelly procedure where a complete detachment of the insertions of the corpora cavernosa from the pubic bones is made, we have not been performing this procedure at all and what we are proposing is separating the corpora in the midline and then mobilizing them laterally. The difference with the Lattimer procedure is that splitting and anchoring of the corpora simply changes the angle of the corpora but does not create a buried entrapped clitoris. We do not have any data supporting results at puberty or adulthood but we believe that by just changing the angle of the corpora, erections will not be painful. We appreciate how you describe our procedure using the words a “simple separation” because that clearly describes our intention to not alter the anatomy and give patient's the potential for normal clitoral erection in adulthood. The difference from the procedure that Dr. Pippi Salle proposed for this conditions is that with our technique a reverting procedure is more feasible in the future.

The Authors
